# To scale or not to scale? Re-engineering biorefining for a more sustainable and circular future

**DOI:** 10.1039/d5su90051d

**Published:** 2025-10-15

**Authors:** Ahmad B. Ghanayem

**Affiliations:** a Department of Chemical Engineering, School of Engineering, The University of Manchester Oxford Road Manchester M13 9PL UK ahmad.ghanayem@manchester.ac.uk

## Abstract

Further to the use of renewable feedstocks, sustainable biorefining requires a holistic process-level approach encompassing techno-economic and life-cycle assessments to help bridge the gap between laboratory innovation and industrial scalability.

## Graphical abstract

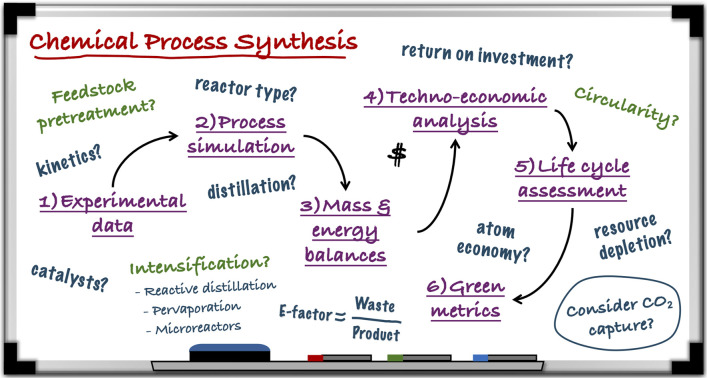

In the face of mounting environmental challenges caused by the inherent unsustainability of the chemical industry, coupled with a rising tide of misinformation regarding climate change, biorefining offers solutions for converting yesterday's waste into tomorrow's wealth. This continuously developing field has gained significant traction thanks to recent international commitments, such as the Paris Agreement, as well as technological advances rooted in the chemical sciences.^1^

In the context of environmental and social concern, various conversion routes have been developed for transforming renewable resources into biofuels and biobased chemicals with reduced carbon footprints compared to their petrochemical-derived counterparts. A well-implemented example of this is the industrial-scale synthesis of biodiesel from the transesterification of triglycerides derived from vegetable oils.^2^

Despite the numerous advantages afforded by biorefining since its large-scale adoption in the late 20th century, significant drawbacks exist in terms of feed pretreatment and downstream separation requirements, which holds true for the majority of chemical processes – biobased or otherwise. Currently, an alarming 10–15% of the world's energy is consumed by distillation processes,^3,4^ which happen to be the technology of choice for around 95% of all industrial separation processes in the chemical industry.^5^ For the biodiesel case, the process usually employs 3 distillation columns, underscoring its dependence on energy-intensive separation techniques.^6^

A common misconception is that the integration of renewable, often waste-derived feedstocks is sufficient to render a process sustainable. While this is a step in the right direction, it must also be acknowledged that the sustainability of a chemical process extends beyond feedstock selection to the consideration of factors such as primary energy demand, choices of solvents (through consideration of their environmental, health and safety profiles) and catalysts (including material criticality), to name a few. This means that, when screening novel synthesis routes, a process-level approach must be applied, with considerations on feasibility through evaluation of the economic and environmental sustainability. This overall philosophy is illustrated in Fig. 1.

**Fig. 1:**
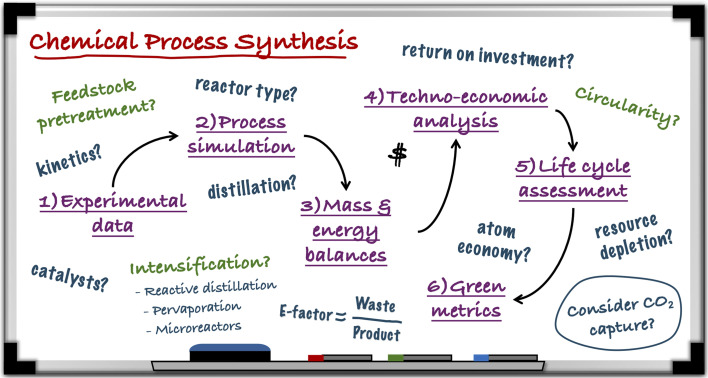
Framework for chemical process development and evaluation.

This approach would ideally begin with the derivation of reaction kinetic data at lab-scale for the chemical/biochemical route in question, which can then be implemented into modelling software such as Aspen Plus® so that the process and all of its streams can be simulated. Additionally, recycle loops should be added to maximise resource utilisation and reduce waste generation. The resulting mass and energy balances can then be used to perform the relevant techno-economic analyses. This is a key task in process appraisal that will provide strong elements for ultimate decision-making and includes performance indicators such as the return on investment, capital/operating expenditures and payback period of the hypothetical chemical plant. If the plant is indeed deemed economically feasible, a thorough life-cycle analysis (LCA) would naturally be the next step for quantifying the environmental impact of all activities involved. Here, the relevant environmental impact categories applied to the framework include global warming potential (GWP), resource and ozone depletion, acidification potential and human toxicity. The mass and energy balances can also be used to calculate additional sustainability metrics rooted in green chemistry.^5^ This could begin with the classic E-factor (ratio of total waste generated to total product produced) and extend to those more recently developed by Sheldon, such as the process mass intensity, reaction mass efficiency and carbon economy.^7^

In addition to considerations directly related to the mass and energy balances of the entire process, the potential for process intensification (PI) can be explored. This is a philosophy employed by chemical engineers that implements innovative design solutions to reduce both the physical and carbon footprints of chemical processes by enhancing mixing and heating or combining unit operations into a single process unit.^1^ One of the most popular examples of PI in practice is reactive distillation (Fig. 2), which combines reaction and separation. The application of this merged operation has been realised in the synthesis of lactic acid (LA), which is mainly used in the food and cosmetics industries, and more recently as a monomer for the production of polylactic acid – a biobased and biodegradable polymer.^8^

**Fig. 2:**
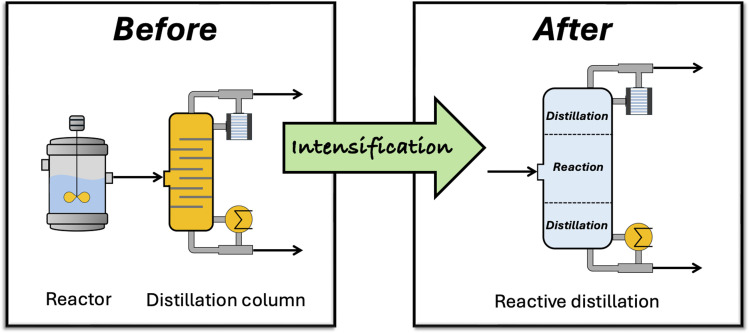
Reactive distillation as a PI technique.

Historically, LA was synthesised from acetaldehyde and hydrogen cyanide. This route has now been widely replaced by biobased processes involving feedstocks such as sugarcane, glycerol and even microalgae. Despite the integration of these renewable substrates, these processes still suffer from costly and energy-intensive downstream separation challenges. In many cases, these account for up to 50% of the total cost of production,^8^ highlighting just some of the real-world challenges typically encountered in biorefining. Reactive distillation technologies could fundamentally change the way these problems are tackled, as demonstrated in a recent paper by Pazmiño-Mayorga *et al.* In their study, a novel design for a reactive distillation column was proposed, enabling simultaneous LA product recovery and impurity removal. When compared to the benchmarked LA processes, the design offers a 13–27% improvement in energy intensity, a 28–32% reduction in material intensity and a 22–36% reduction in overall water consumption. CO_2_ emissions also saw a reduction of up to 33%.^9^

The LA example is a perfect illustration of why feedstock sustainability alone is not necessarily enough when designing greener chemical processes. Overlooking key factors such as separation requirements, energy/material intensity and total emissions can result in uninformed judgements leading to deceptively unsustainable chemical processes. This reinforces the argument that a process-level approach must be undertaken in the early stages of synthetic route evaluation – one grounded in rigorous simulations, detailed LCAs and sustainability metrics including GWP, primary energy demand, resource depletion and freshwater consumption. In addition, it highlights the need for close collaboration between chemists and chemical engineers, enabling them to synergise their strengths across the multiple scales to be considered in process design all the way from molecular level to plant level.^10^

In addition to PI, another potential way to reduce process energy demands is by replacing chemocatalysts with biocatalysts, which typically require milder reaction conditions, thus eliminating the need for the high temperatures and pressures usually required for conventional chemical conversions. Despite reaction rates tending to be lower compared to chemocatalysts, they generally offer much higher selectivities.^11^ The high cost traditionally associated with enzyme purification however (up to 80% of total enzyme production cost)^12^ is yet another example of why biobased manufacturing routes can rarely operate at the profit margins expected in the bulk chemicals sector.^13^ Despite this, recent advances in the field of biocatalysis could provide a much-needed push towards scalability, with a noteworthy example being the enzymatic synthesis of styrene.^14^

Styrene is an important commodity petrochemical that finds its main use in the plastics sector as a monomer, for example in the production of polystyrene. Over 31 million tonnes of this monomer were produced in 2018, with an associated CO_2_ release exceeding 24 million tonnes.^15,16^ The fossil-based production of styrene involves the dehydrogenation of ethylbenzene using superheated steam over an iron(iii) oxide catalyst. This process suffers from very demanding operational conditions, such as high temperature requirements (540–650 °C) and equilibrium limitations, the latter of which necessitate the use of large quantities of superheated steam (around 3 tons per ton of ethylbenzene),^15^ making it one of the most energy-intensive commodity petrochemical production processes worldwide.^17^

Fortunately, styrene can also be synthesised naturally in certain types of bacteria and fungi through an enzyme-catalysed reaction starting from the renewable amino acid l-phenylalanine, which can be derived from lignocellulosic biomass or glucose.^18^ The main advantage of this route is the use of significantly milder operating conditions (30–37 °C and atmospheric pressure).^14^ Additionally, the clever use of whole microbial cells rather than purified enzymes eliminates the costs associated with enzyme purification, with the cells essentially acting as microscopic chemical reactors, as depicted in Fig. 3. Messiha *et al.* recently demonstrated the viability of this route as a competitive alternative to the conventional ethylbenzene pathway.^14^

**Fig. 3:**
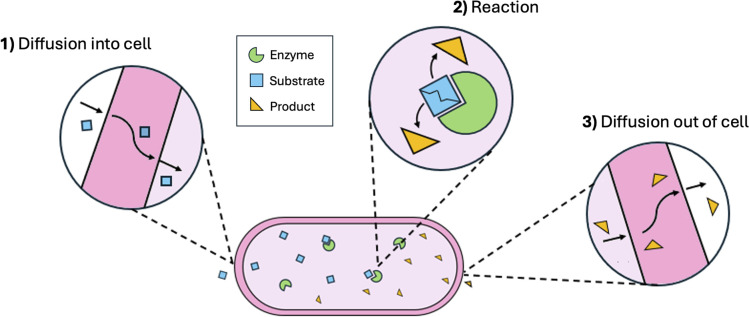
Illustration of whole-cell biocatalysis.

The latest developments in biocatalytic styrene production have generated great industrial interest, with a notable example being a recent 5-year 9 million pound partnership between Shell, the University of Manchester and the UK Government, in which the author of this essay is currently taking part as a PhD student. This project, entitled “Sustainable Commodity Chemicals through Enzyme Engineering and Design” (SuCCEED, BB/Y003276/1),^13^ aims to bring biobased chemicals, including styrene, to market with an interdisciplinary team of biologists, chemists and chemical engineers working to develop the next generation of biorefineries. It also highlights the importance of meaningful collaborations between academia and industry to address sustainability issues in the chemical industry.

While it is impossible to overlook the fact that biorefining has immense potential in reforming the way our chemical industry operates, there is much to be done to address its limitations and inefficiencies. This requires close collaboration between scientists, industrialists and government officials to bridge the gap between laboratory innovation and industrial scalability. A strong emphasis must be placed on the evaluation of economic and environmental performance indicators to make informed decisions on the scale-up and implementation of processes. Essentially, biorefining must be rebranded from a simple green-feedstock swap to a full-scale reimagination of how we tackle our sustainability challenges before we reach a critical environmental tipping point.

## Conflicts of interest

There are no conflicts to declare.

## Data Availability

There is no additional data associated with this article.
